# An Evaluation of Perinatal Human Immunodeficiency Virus Transmissions in New York State, 2007–2020

**DOI:** 10.1097/og9.0000000000000212

**Published:** 2026-10-20

**Authors:** Carol-Ann Swain, Calvin Wong, Amanda Stolz, Sarah Macinski, Lisa Haskin, Suzanne Kaufman

**Affiliations:** New York State Department of Health, Albany, New York.

## Abstract

**OBJECTIVE:**

To evaluate predictors of perinatal human immunodeficiency virus (HIV) transmission and birthing parent unsuppressed HIV viral load proximate to delivery in New York State.

**METHODS:**

This was a population-based, retrospective cohort study of perinatal HIV transmission from 2007 to 2020 using data from the New York State Department of Health's Wadsworth Center, HIV Surveillance System, and medical record abstraction. Birthing parents living with HIV and their live-born infants were included. Multivariable models estimated the association between predictors and perinatal HIV transmission with adjusted odds ratios (AORs) and 95% CIs reported. Risk ratios (RRs) were calculated to evaluate predictors of an unsuppressed viral load before (closest test results) delivery.

**RESULTS:**

Of 5,722 birthing parent–infant dyads included in analysis, perinatal transmission was presumed or confirmed for 44 infants (0.77%). In multivariable analyses, an unsuppressed viral load at the test closest to delivery predicted perinatal HIV transmission (AOR 10.47, 95% CI, 4.35–27.80). The strongest predictors of an unsuppressed viral load in multivariable analyses were documented illicit substance use during pregnancy (adjusted RR 1.79, 95% CI, 1.57–2.03), nonuse of prenatal antiretroviral therapy during pregnancy (adjusted RR 2.91, 95% CI, 2.46–3.45), and later initiation of prenatal care (second compared with first trimester: adjusted RR 1.34, 95% CI, 1.17–1.54; third trimester: adjusted RR 1.81, 95% CI, 1.51–2.18).

**CONCLUSION:**

Rates of perinatal HIV transmission were low in this retrospective cohort study. However, unsuppressed viral load proximate to delivery remains a strong predictor for perinatal transmission, providing insight into priorities for addressing rare but ongoing perinatal HIV transmission.

## Abstract 2

In this retrospective cohort study in New York State from 2007 to 2020, perinatal human immunodeficiency virus transmission was low (<1%), but birthing parent unsuppressed viral load proximate to delivery remained a key predictor for transmission.

Within the United States, perinatal transmission of human immunodeficiency virus (HIV) peaked in the 1990s with more than 1,600 transmissions in 1991.^1^ Since then, transmissions have declined as a result of improvements in HIV drug therapies and policies that support HIV testing during pregnancy and on labor and delivery units.^2,3^ New York State achieved the Centers for Disease Control and Prevention goal of eliminating perinatal transmission (ie, less than one HIV infection per 100,000 live births and infection in less than 1% of HIV-exposed infants)^4^ in 2013 and 2015–2023.^5^ Although perinatal transmissions are rare, ongoing assessment of associated factors is critical to ensure that transmissions do not rebound.^6,7^

Strong associations exist between structural and behavioral factors and perinatal HIV transmissions. These factors include inadequate prenatal care and lack of access or nonadherence to antiretroviral therapy (ART) during pregnancy or postpartum (if breastfeeding/chestfeeding). A major finding from a 2002–2006 New York State investigation was that HIV acquired during pregnancy increased the risk of perinatal transmission.^8^ Since then, electronic laboratory reporting was implemented, testing technologies and ART efficacy were improved, and New York State adopted the U.S. federal recommendation to repeat HIV testing in the third trimester in high-incidence areas to identify seroconversion during pregnancy.^3^ Given these changes and the strong association between high viremia during pregnancy and perinatal transmission,^9^ the two aims of the present study were to 1) evaluate predictors of perinatal HIV transmission in New York State in a modern period and 2) evaluate predictors of an unsuppressed viral load proximate to delivery.

## METHODS

Deliveries with at least one live-born infant in New York State from January 1, 2007, to December 31, 2020, among birthing parents living with HIV were eligible for inclusion in this retrospective cohort study. To ensure that each delivery event was represented once, only one infant was retained for analysis from deliveries of multifetal gestations; if discordant HIV diagnoses between exposed infants occurred, the infected infant was retained. Delivery events were also excluded from analyses if 1) no medical record information was available, 2) infants had unknown HIV infection status (defined per the HIV surveillance case definition),^10^ or 3) the birthing parent was diagnosed after delivery and the infant had a negative Newborn Screening Program result, indicating no HIV exposure before birth.

Deliveries were identified from the New York State Department of Health Wadsworth Center Newborn Screening Program, with supplemental case finding from the New York State Department of Health Wadsworth Center Bloodborne Viruses Laboratory Pediatric HIV Testing Service and other New York State Permitted Clinical Laboratories that report the results of all HIV-related tests for individuals who reside in or receive HIV-related care in New York State to the New York State Department of Health.^11,12^ Data on HIV-exposed delivery events were collected as part of routine HIV surveillance and regulatory monitoring, which includes medical record abstraction by the Island Peer Review Organization. Data on birthing parent HIV viral load testing were extracted from the New York State Department of Health HIV Surveillance System. These activities are a part of comprehensive regulatory oversight and routine public health HIV surveillance and therefore exempt from IRB review.

All infants born in New York State, unless there is written parental religious objection, are screened by the Newborn Screening Program for in utero exposure to HIV within 24–36 hours of birth.^13^ Between 2007 and 2010, HIV-1 antibodies for infants born to fully seroconverted birthing parents were detected with an enzyme immunoassay with Western blot confirmation for reactive tests. Beginning in January 2011, a Microelisa system was used for the qualitative detection of antibodies to HIV-1. Western blot confirmation was discontinued in 2015. In December 2018, the program began using a microsphere immunoassay with the Geenius assay, a rapid immunochromatographic test for the detection of antibodies to HIV-1 and HIV-2, for confirmation.

From 1995 to October 2008, the New York State Department of Health Wadsworth Center Bloodborne Viruses Laboratory Pediatric HIV Testing Service used a laboratory-developed HIV-1 DNA polymerase chain reaction (PCR) for infant diagnostic testing.^14^ The laboratory switched to HIV-1 qualitative RNA testing in November 2008 and added HIV-2 qualitative RNA testing for HIV-2–exposed infants in 2010. Together, qualitative HIV nucleic acid (RNA or DNA) testing results reported by the New York State Department of Health Wadsworth Center Bloodborne Viruses Laboratory Pediatric HIV Testing Service and New York State Permitted Clinical Laboratories were used to determine the HIV infection status for exposed infants.

Perinatal HIV transmission and an unsuppressed viral load proximate to but preceding birth were the outcomes of interest. Confirmed perinatal HIV transmission was defined as an infant with a positive qualitative nucleic acid test followed by a second positive qualitative nucleic acid test on a specimen collected on a different day. Presumed perinatal transmission was defined as an infant with a single documented positive qualitative nucleic acid test with no subsequent negative qualitative nucleic acid test. Absence of HIV infection was confirmed if an infant had at least two negative HIV qualitative nucleic acid tests at or after 4 weeks of age, with at least one of those tests being at or after 4 months of age. An infant was presumed to be uninfected if they had at least one negative qualitative nucleic acid test at or after 4 weeks of age and no documented positive nucleic acid tests.^10^ For this analysis, presumed infant HIV infection and confirmed infant HIV infection were combined. Birthing parent unsuppressed viral load was defined as an HIV viral load of 200 copies/mL or more for the test result during pregnancy that was closest to but before delivery.

Study variables included demographic, behavioral, and clinical factors. Demographic factors were year of delivery, region of residence, birthing parent race and ethnicity (defined by the National HIV Surveillance System),^15^ and birthing parent age at delivery. Behavioral factors were whether an infant was breastfed or chestfed and documented illicit substance use by the birthing parent during pregnancy. Illicit substance use included depressants, hallucinogens, opioids, and stimulants. Clinical factors were timing of birthing parent HIV diagnosis, sexually transmitted infection diagnosis during pregnancy, delivery type, length of membrane rupture, birth weight, ART (prenatal, labor and delivery, and newborn), trimester prenatal care was initiated, and unsuppressed viral load proximate to delivery.

All study covariates were selected a priori on the basis of established literature; a sufficient component cause approach was used to further reduce redundancy among covariates.^16^ No adjustment for multiple comparisons was applied. The crude odds ratio (OR) and 95% CI between each covariate and perinatal HIV transmission were calculated in bivariate analyses. Covariates that were significant (α level of 0.05) in bivariate analyses were included in the multivariable logistic regression model to calculate adjusted ORs (AORs) and 95% CIs. All ORs for perinatal transmission were calculated with exact methods. Infant breastfed/chestfed was excluded from multivariable analysis because it was documented in only two infants, rendering the AOR inestimable. The three birthing parents who were diagnosed at delivery or after their child's birth were excluded from multivariable analysis because they also rendered the AOR inestimable. We excluded ART because of its direct effect on viral suppression. Instead, the model included viral suppression because it is a key factor linking ART use and perinatal transmission.

To evaluate laboratory-based inclusion/exclusion criteria, we conducted sensitivity analyses including 477 delivery events previously excluded for incomplete chart review or unknown infant diagnosis status. These delivery events were assigned perinatal transmission probabilities under two assumptions: 1) a transmission “rate” (0.77%) equal to that observed among included infants and 2) a more conservative “rate” (1.54%) of twice that observed. In a separate analysis, exact ORs and CIs were recalculated after the exclusion of previously included presumed negative infants (n=1,233).

Predictors of having an unsuppressed viral load proximate to delivery were evaluated using crude risk ratios (RRs) and 95% CIs in bivariate analyses. Birthing parents who were diagnosed at delivery or after their child's birth were excluded from the multivariable analysis. Multiple imputation, implemented with SAS Proc MI, was used to address missing data for study covariates included in the multivariable model for unsuppressed viral load. Nineteen regression imputation cycles were performed for each variable imputed on the basis of the literature, which suggests that the number of imputations should be equal to or greater than the variable with the highest fraction of missing information.^17^ Adjusted RRs with 95% CIs were calculated with a multivariable Poisson regression model with robust variance and included only covariates that were significant (α level of 0.05) in bivariate analyses.

All analyses were conducted with SAS 9.4.

## RESULTS

Of the 3,294,473 live births that occurred between 2007 and 2020 in New York State,^18^ 6,342 infants were born to pregnant people living with HIV (HIV exposure birth rate 0.19%). A total of 5,722 HIV-exposed deliveries were retained for analysis (Fig. 1). Birthing parents living with HIV were predominantly Black or African American, non-Hispanic (60.9%), 20–39 years old at delivery (20–29 years 41.1%, 30–39 years 49.0%), diagnosed with HIV before pregnancy (85.8%), and virally suppressed at the test closest to but before delivery (78.5%; median 15 days, interquartile range 5–28 days) (Table 1); 89% of birthing parents had a test within 60 days (74.5% 0–30 days n=4,263; 14.8% 31–60 days n=851) of delivery, and 2.5% (n=144) had a last test 90 days or more before delivery.

**Fig. 1.**
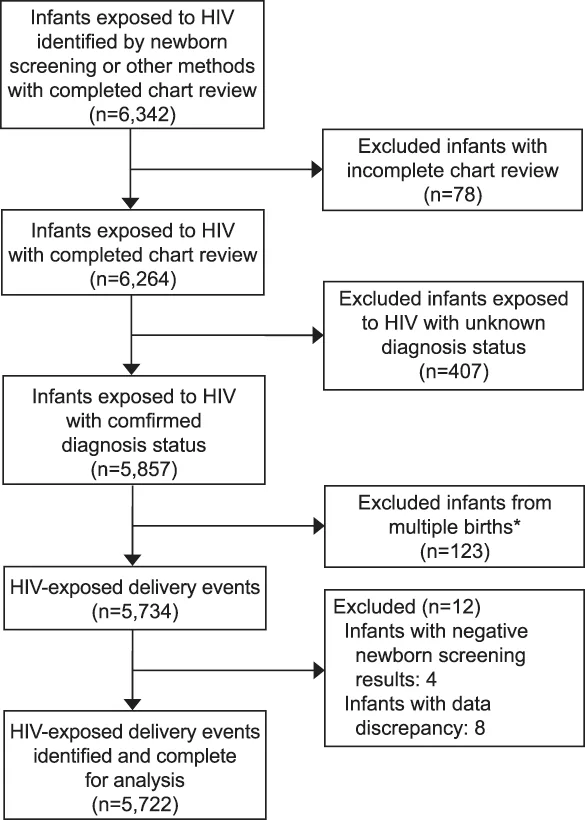
Inclusion and exclusion criteria for human immunodeficiency virus (HIV)–exposed delivery events. *Only one infant was retained from deliveries with multiple gestation.

*Swain. New York State Perinatal HIV, 2007–2020. O&G Open 2026*.

**Table 1.** Bivariate Analysis of Human Immunodeficiency Virus–Exposed Deliveries and Perinatal Human Immunodeficiency Virus Transmission in New York State, 2007–2020

Characteristics	HIV-Exposed Delivery Events, n (column %)	Perinatal HIV Transmission	
		n (row %)	Crude OR (95% CI)
Total	5,722 (100)	44 (0.77)	
Year of delivery*			**0.86 (0.79–0.94)**
2007	552 (9.7)	7 (1.3)	
2008	475 (8.3)	6 (1.3)	
2009	503 (8.8)	9 (1.8)	
2010	461 (8.1)	3 (0.7)	
2011	463 (8.1)	6 (1.3)	
2012	395 (6.9)	2 (0.5)	
2013	425 (7.4)	2 (0.5)	
2014	386 (6.8)	3 (0.8)	
2015	369 (6.5)	—	
2016	390 (6.8)	2 (0.5)	
2017	369 (6.5)	1 (0.3)	
2018	308 (5.4)	—	
2019	327 (5.7)	2 (0.6)	
2020	299 (5.2)	1 (0.3)	
Region of residence			
New York City	4,395 (76.8)	37 (0.8)	1.56 (0.69–4.17)
New York State†	1,296 (22.7)	7 (0.5)	Reference
Out of state	28 (0.5)		
Missing	3 (0.1)		
Race and ethnicity			
Black or African American	3,487 (60.9)	20 (0.6)	0.63 (0.21–2.56)
Hispanic or Latino	1,562 (27.3)	20 (1.3)	1.42 (0.47–5.76)
Other‡ or multiple races, non-Hispanic	225 (3.9)	—	
White	443 (7.7)	4 (0.9)	Reference
Missing	5 (0.1)		
Age at delivery (y)			
Younger than 20	235 (4.1)	3 (1.3)	1.31 (0.25–4.39)
20–29	2,354 (41.1)	23 (1.0)	Reference
30–39	2,801 (49.0)	17 (0.6)	0.62 (0.31–1.21)
40 and older	327 (5.7)	1 (0.3)	0.31 (0.01–1.93)
Missing	5 (0.1)		
Timing of HIV diagnosis			
Before pregnancy	4,907 (85.8)	29 (0.6)	Reference
During pregnancy	748 (13.1)	12 (1.6)	**2.74 (1.27–5.57)**
At delivery or after child's birth	61 (1.1)	3 (4.9)	**8.69 (1.65–29.27)**
Missing	6 (0.1)		
Illicit substance use during pregnancy			
No documented use	4,857 (84.9)	25 (0.5)	Reference
Documented use	809 (14.1)	18 (2.2)	**4.40 (2.25–8.43)**
Missing	56 (1.0)	1 (1.8)	
STI diagnosis during pregnancy			
No documented diagnosis	3,706 (64.8)	28 (0.8)	Reference
Documented diagnosis	2,016 (35.2)	16 (0.8)	1.05 (0.53–2.02)
Delivery type			
Cesarean	3,085 (53.9)	27 (0.9)	Reference
Vaginal	2,607 (45.6)	17 (0.7)	0.74 (0.38–1.42)
Missing	30 (0.5)		
Length of membrane rupture			
Less than 2 h	1,251 (21.9)	10 (0.8)	Reference
2 h–4 h	420 (7.3)	2 (0.5)	0.59 (0.06–2.8)
More than 4 h	1,263 (22.1)	8 (0.6)	0.79 (0.27–2.24)
Cesarean	2,337 (40.8)	19 (0.8)	1.02 (0.45–2.46)
Missing	451 (7.9)	5 (1.1)	
Neonatal birth weight			
2,500 g or more	4,748 (83)	34 (0.7)	Reference
Less than 2,500 g	958 (16.7)	10 (1.0)	1.46 (0.64–3.04)
Missing	16 (0.3)		
Antiretroviral therapy			
Prenatal			
Yes	5,427 (94.8)	26 (0.5)	Reference
No	222 (3.9)	14 (6.3)	**13.96 (6.64–28.21)**
Missing	73 (1.3)	4 (5.5)	
Labor and delivery			
Yes	4,713 (82.4)	33 (0.7)	Reference
No	955 (16.7)	11 (1.2)	1.65 (0.75–3.37)
Missing	54 (0.9)		
Newborn			
Yes	5,669 (99.1)	41 (0.7)	Reference
No	35 (0.6)	3 (8.6)	**12.85 (2.42–43.69)**
Missing	18 (0.3)	0	
Infant breastfed/chestfed			
No	5,682 (99.3)	42 (0.7)	Reference
Yes	30 (0.5)	2 (6.7)	**9.58 (1.07–40.13)**
Missing	10 (0.2)		
Trimester prenatal care began			
1st	2,434 (42.5)	13 (0.5)	Reference
2nd	1,765 (30.9)	15 (0.8)	1.60 (0.71–3.66)
3rd	432 (7.6)	3 (0.7)	1.30 (0.24–4.77)
Missing	1,091 (19.1)	13 (1.2)	
Viral load suppression at test closest to delivery			
Median 15 d, IQR 5–28 d			
Suppressed	4,493 (78.5)	8 (0.2)	Reference
Unsuppressed	961 (16.8)	23 (2.4)	**13.74 (5.91–35.63)**
Missing	268 (4.7)	13 (4.9)	

OR, odds ratio; HIV, human immunodeficiency virus; STI, sexually transmitted infection; IQR, interquartile range.

Bold entries indicate statistically significant values.

* Assessed as a continuous (ordinal) predictor.

† New York State, excluding New York City.

‡ American Indian or Alaska Native, Asian, Native Hawaiian or Other Pacific Islander, or categorized as “other race” in the medical record.

Perinatal HIV transmission was documented in 44 infants (0.77%); of these, 41 were confirmed positive with at least two positive nucleic acid tests, and three were presumed positive with one positive nucleic acid test. Absence of perinatal HIV transmission was presumed in 1,233 infants and confirmed in 4,445 infants (not shown).

In bivariate analyses, year of delivery (OR 0.86, 95% CI, 0.79–0.94), birthing parent diagnosed with HIV during pregnancy (OR 2.74, 95% CI, 1.27–5.57) or at or after delivery (OR 8.69, 95% CI, 1.65–29.27), documented illicit substance use during pregnancy (OR 4.40, 95% CI, 2.25–8.43), nonuse of ART in the prenatal (OR 13.96, 95% CI, 6.64–28.21) and newborn (OR 12.85, 95% CI, 2.42–43.69) periods, infant was breastfed/chestfed (OR 9.58, 95% CI, 1.07–40.13), and an unsuppressed HIV viral load at the test closest to delivery (OR 13.74, 95% CI, 5.91–35.63) were predictors of perinatal HIV transmission (Table 1). It is important to note that of the eight suppressed individuals who transmitted, seven had a documented viral load of more than 5,000 copies/mL during pregnancy. In multivariable analyses, an unsuppressed viral load proximate to delivery remained a significant predictor of transmission after adjustment for year of delivery, timing of birthing parent HIV diagnosis, and illicit substance use during pregnancy (AOR 10.47, 95% CI, 4.35–27.80) (Table 2).

**Table 2.** Multivariable Regression Model Predicting Perinatal Human Immunodeficiency Virus Transmission in New York State, 2007–2020 (n=5,411)

Characteristics	Adjusted OR (95% CI)
Year of delivery*	0.92 (0.82–1.01)
Timing of birthing parent HIV diagnosis	
Before pregnancy	Reference
During pregnancy	2.00 (0.76–4.73)
Illicit substance use during pregnancy	
No documented use	Reference
Documented use	2.18 (0.92–4.67)
Viral load suppression at test closest to delivery	
Suppressed	Reference
Unsuppressed	**10.47 (4.35–27.80)**

OR, odds ratio; HIV, human immunodeficiency virus.

Bold entries indicate statistically significant values.

* Assessed as a continuous (ordinal) predictor.

Introducing previously excluded records (n=477) under the assumption of an equal or double transmission rate resulted in an estimated four and eight additional infected infants, respectively. However, a review of New York State Department of Health HIV Surveillance System data from 2007 to May 2025 did not identify additional diagnoses among anyone born perinatally exposed in New York State. Limiting the original analyses to confirmed negatives did not substantively alter study findings (Appendices 1 and 2, available online at https://links.lww.com/AOG/F27).

The strongest predictors of having an unsuppressed viral load at the test closest to delivery in bivariate analyses were nonuse of ART in the prenatal period, birthing parent diagnosed with HIV at or after delivery, prenatal care beginning in the third trimester, and documented illicit substance use during pregnancy (Table 3). Year of delivery (adjusted RR 0.93, 95% CI, 0.92–0.95), older birthing parent age at delivery (30–39 years: adjusted RR 0.67, 95% CI, 0.59–0.76; 40 years or older: adjusted RR 0.75, 95% CI, 0.58–0.97), illicit substance use during pregnancy (adjusted RR 1.79, 95% CI, 1.57–2.03), nonuse of prenatal ART during pregnancy (adjusted RR 2.91, 95% CI, 2.46–3.45), and later trimesters of prenatal care initiation (second trimester: adjusted RR 1.34, 95% CI, 1.17–1.54; third trimester: adjusted RR 1.81, 95% CI, 1.51–2.18) remained associated with an unsuppressed viral load at test closest to delivery in multivariable analyses (Table 4).

**Table 3.** Bivariate Analysis of Human Immunodeficiency Virus–Exposed Delivery Events and Unsuppressed Viral Load at Test Closest to Delivery in New York State, 2007–2020

Characteristics	HIV-Exposed Delivery Events, n (column%)	Unsuppressed Viral Load at Test Closest to Delivery	
		n (row%)	Crude RR (95% CI)
Total	5,722 (100)	961 (16.8)	
Year of delivery*			**0.92 (0.91–0.94)**
2007	552 (9.7)	140 (25.4)	
2008	475 (8.3)	112 (23.6)	
2009	503 (8.8)	106 (21.1)	
2010	461 (8.1)	90 (19.5)	
2011	463 (8.1)	83 (17.9)	
2012	395 (6.9)	67 (17.0)	
2013	425 (7.4)	69 (16.2)	
2014	386 (6.8)	49 (12.7)	
2015	369 (6.5)	61 (16.5)	
2016	390 (6.8)	42 (10.8)	
2017	369 (6.5)	49 (13.3)	
2018	308 (5.4)	27 (8.8)	
2019	327 (5.7)	40 (12.2)	
2020	299 (5.2)	26 (8.7)	
Region of residence			
New York City	4,395 (76.8)	773 (17.6)	**1.22 (1.06–1.42)**
New York State†	1,296 (22.7)	185 (14.3)	Reference
Out of state	28 (0.5)	3 (10.7)	0.91 (0.31–2.62)
Missing	3 (0.1)		
Race and ethnicity			
Black or African American	3,487 (60.9)	605 (17.4)	**1.30 (1.02–1.67)**
Hispanic or Latino	1,562 (27.3)	267 (17.1)	1.29 (0.99–1.68)
Other‡ or multiple races, non-Hispanic	225 (3.9)	31 (13.8)	1.04 (0.69–1.55)
White	443 (7.7)	58 (13.1)	Reference
Missing	5 (0.1)		
Age at delivery (y)			
Younger than 20	235 (4.1)	64 (27.2)	**1.34 (1.07–1.67)**
20-29	2,354 (41.1)	471 (20.0)	Reference
30-39	2,801 (49)	377 (13.5)	**0.66 (0.59–0.75)**
40 and older	327 (5.7)	49 (15.0)	**0.74 (0.56–0.97)**
Missing	5 (0.1)		
Timing of HIV diagnosis			
Before pregnancy	4,907 (85.8)	796 (16.2)	Reference
During pregnancy	748 (13.1)	157 (21.0)	**1.32 (1.13–1.53)**
At delivery or after child's birth	61 (1.1)	8 (13.1)	**3.66 (2.37–5.65)**
Missing	6 (0.1)		
Illicit substance use during pregnancy			
No documented use	4,857 (84.9)	711 (14.6)	Reference
Documented use	809 (14.1)	244 (30.2)	**2.12 (1.87–2.39)**
Missing	56 (1.0)	6 (10.7)	
STI diagnosis during pregnancy			
No documented diagnosis	3,706 (64.8)	598 (16.1)	Reference
Documented diagnosis	2,016 (35.2)	363 (18.0)	1.08 (0.96–1.22)
Antiretroviral therapy			
Prenatal			
Yes	5,427 (94.8)	860 (15.8)	Reference
No	222 (3.9)	90 (40.5)	**4.68 (4.15–5.27)**
Missing	73 (1.3)	11 (15.1)	
Trimester prenatal care began			
1st	2,434 (42.5)	294 (12.1)	Reference
2nd	1,765 (30.9)	331 (18.8)	**1.57 (1.36–1.81)**
3rd	432 (7.6)	122 (28.2)	**2.37 (1.98–2.85)**
Missing	1,091 (19.1)	214 (19.6)	

RR, risk ratio; HIV, human immunodeficiency virus; STI, sexually transmitted infection.

Bold entries indicate statistically significant values.

* Assessed as a continuous (ordinal) predictor.

† New York State, excluding New York City.

‡ American Indian or Alaska Native, Asian, Native Hawaiian or Other Pacific Islander, or categorized as “other race” in the medical record.

**Table 4.** Multivariable Regression Model Predicting Unsuppressed Viral Load at Test Closest to Delivery in New York State for the Imputed Sample (n=5,441, Number of Imputations=19), 2007–2020

Characteristics	Adjusted RR (95% CI)
Year of delivery*	**0.93 (0.92–0.95)**
Region	
New York City	1.15 (0.99–1.33)
New York State†	Reference
Out of state	1.17 (0.44–3.13)
Race and ethnicity	
Black or African American, non- Hispanic	**1.29 (1.02–1.66)**
Hispanic or Latino	1.25 (0.99–1.61)
Other‡ or multiple races, non-Hispanic	1.17 (0.79–1.72)
White, non-Hispanic	Reference
Birthing parent age at delivery (y)	
Younger than 20	1.17 (0.94–1.45)
20–29	Reference
30–39	**0.67 (0.59–0.76)**
40 or older	**0.75 (0.58–0.97)**
Timing of birthing parent HIV diagnosis	
Before pregnancy	Reference
During pregnancy	1.06 (0.91–1.24)
Illicit substance use during pregnancy	
No documented use	Reference
Documented use	**1.79 (1.57–2.03)**
Antiretroviral medication	
Prenatal	
Yes	Reference
No	**2.91 (2.46–3.45)**
Trimester prenatal care began	
1st	Reference
2nd	**1.34 (1.17–1.54)**
3rd	**1.81 (1.51–2.18)**

RR, risk ratio; HIV, human immunodeficiency virus.

Bold entries indicate statistically significant values.

* Assessed as a continuous (ordinal) predictor.

† New York State, excluding New York City.

‡ American Indian or Alaska Native, Asian, Native Hawaiian or Other Pacific Islander, or categorized as “other race” in the medical record.

## DISCUSSION

In this retrospective cohort study, perinatal HIV transmission occurred infrequently (0.77%; n=44) among HIV-exposed infants in New York State. In multivariable analyses, an unsuppressed viral load proximate to delivery was associated with perinatal HIV transmissions. We observed that 17% of birthing parents (n=961) had an unsuppressed viral load at the test closest to but before delivery, and this group accounted for about half (52.3%) of perinatal HIV transmissions. Identified predictors for an unsuppressed viral load proximate to delivery included year of delivery, older birthing parent age, illicit substance use during pregnancy, nonuse of ART in pregnancy, and later initiation of prenatal care.

Later initiation of prenatal care may reduce the likelihood of birthing parent viral suppression by impeding timely ART initiation and adherence support or receipt of other supportive services.^19,20^ These mechanisms were not directly addressed in this study and require further investigation.

The predictors of an unsuppressed viral load in the present study, ie, prenatal illicit substance use (40.9% of transmissions) and inadequate prenatal care (28.2% of transmissions), align with predictors of perinatal transmission observed in a previous New York State evaluation, which reported increased transmission when HIV was acquired during pregnancy (ie, high viral load: AOR 15.19, 95% CI, 3.98–56.30), with prenatal illicit substance use (AOR 2.66, 95% CI, 1.33–5.27), and with inadequate prenatal care (0–2 visits: AOR 2.37, 95% CI, 1.11–4.91).^8^

Although birthing parent HIV viral load is a key determinant of transmission risk during pregnancy, delivery, and breastfeeding/chestfeeding, transmission is not inevitable even with high viremia.^9^ Most pregnancies do not result in transmission because multiple clinical and biologic factors such as mode of delivery and newborn ART influence risk. However, the persistence of high viremia, substance use, and late/limited prenatal care in, albeit rare, perinatal transmissions underscore the importance of prepregnancy and interpregnancy counseling and care. Findings suggest these may be unmet needs for birthing people living with or at risk for HIV. These time periods are critical to identify and treat chronic diseases, engage partners in HIV testing, support reproductive life planning, including early and consistent prenatal care, and address unmet health-related social needs.^21,22^

Two transmissions occurred among individuals with documented breastfeeding/chestfeeding. Infant feeding choices and practices are strongly influenced by interpersonal, community, organizational, and environmental factors, including socioeconomic status, health care professional interactions, and cultural and gender norms.^23^ The decision to breastfeed/chestfeed is especially relevant given the 2023 update to the U.S. perinatal guidelines, which support breastfeeding/chestfeeding for individuals living with HIV in the United States who are on ART with sustained viral load suppression.^24^ Strategies that do not address intersecting barriers in relation to infant feeding could lead to increased transmission rates.^25^

Incorporating previously excluded records in sensitivity analyses indicated that four and potentially eight additional infected infants may have been missed, indicating some susceptibility to extreme assumptions. However, additional diagnoses among individuals born perinatally exposed within New York State were not identified, indicating that large deviations from observed transmission patterns were unlikely.

We note the following limitations. Perinatal transmission was rare during the study period. Small cell sizes limited statistical power and resulted in wide CIs, indicating imprecision in some point estimates, which should be interpreted with caution. We used viral load at the test closest to but before delivery to define birthing parent unsuppressed viral load. However, this single viral load measurement may not reflect the true transmission risk because it does not fully capture the duration or magnitude of viremia during pregnancy. In addition, our analysis may not fully capture residual confounding because of changes in HIV testing methods for infants and birthing parents. However, improvements in laboratory testing sensitivity and screening practices would reduce rather than increase the likelihood of missed identification of HIV exposure or transmission.^26^ Despite these limitations, the findings are consistent in direction and magnitude with prior literature.^8^

In conclusion, perinatal HIV transmission was low, but an unsuppressed viral load proximate to delivery remains a persistent risk factor for transmission. To maintain low levels of perinatal HIV transmission, we must address syndemics, prepregnancy and interpregnancy care, health-related social needs, and social, structural, and behavioral factors. Models of prenatal care and reproductive health service delivery should span the life course and be attuned to individual needs, cultural expectations, and social determinants of health (eg, employment, transportation, caregiving responsibilities).^22^
